# Combined associations of vitamin D and cognitive function with all-cause mortality among older adults in Chinese longevity areas: A prospective cohort study

**DOI:** 10.3389/fpubh.2023.1024341

**Published:** 2023-05-02

**Authors:** Miao Dai, Quhong Song, Xiang Wang, Ying Li, Taiping Lin, Rui Liang, Tingting Jiang, Xiaoyu Shu, Ning Ge, Jirong Yue

**Affiliations:** ^1^Department of Geriatrics, Jiujiang First People’s Hospital, Jiujiang, Jiangxi, China; ^2^Department of Geriatrics and National Clinical Research Center for Geriatrics, West China Hospital of Sichuan University, Chengdu, Sichuan, China; ^3^Department of Cardiology, Jiujiang First People’s Hospital, Jiujiang, Jiangxi, China

**Keywords:** vitamin D, cognition, older adults, all-cause mortality, a cohort study

## Abstract

**Objectives:**

While both vitamin D deficiency and cognitive impairment have individually been linked to a greater risk of all-cause mortality, the combined effects of these two different conditions have not previously been explored in this context. We aimed to investigate the combined impact of vitamin D concentration and cognitive impairment on all-cause mortality in older adults.

**Methods:**

The analyzed data were collected from community-dwelling adults ≥65 years of age that were enrolled in the Chinese Longitudinal Healthy Longevity Survey (*n* = 1,673). The Mini-Mental Status Examination (MMSE) was used to assess cognitive function, while the plasma 25-hydroxyvitamin D [25(OH)D] test was used to assess vitamin D status. The associations between vitamin D concentration, cognitive function, and all-cause mortality were assessed with Cox proportional hazards models. We used restricted cubic splines to examine the dose–response relationship between vitamin D and the risk of all-cause mortality and used joint effect testing to explore interactions between vitamin D concentration and cognitive function.

**Results:**

During a mean (SD) follow-up of 3.8 (1.9) years, 899 (53.7%) deaths occurred. A negative dose–response relationship was observed between 25(OH)D concentration and cognition impairment at baseline, as well as the odds of all-cause mortality during follow-up. Similarly, cognitive impairment was significantly related to all-cause mortality risk (HR 1.81, 95% CI: 1.54 to 2.12). The combined analyses showed positive associations, with the highest mortality risk observed in older adults with both low vitamin D and cognitive impairment (HR 3.04, 95% CI: 2.40 to 3.86). Moreover, the interaction between 25(OH)D concentration and cognitive function was found to be significant in relation to the risk of mortality (*p* for interaction <0.001).

**Conclusion:**

Lower plasma 25(OH)D and cognitive impairment were, respectively, associated with increased all-cause mortality risks. The 25(OH)D concentration and cognitive impairment exhibited a combined additive effect on all-cause mortality among older Chinese adults.

## Introduction

The global population is aging at an unprecedented rate, with an estimated 1.5 billion people projected to be aged 65 and over by 2050 (1). With this demographic shift, there is a growing concern for the health and well-being of older adults, including the risk of all-cause mortality. Cognitive function and vitamin D have been identified as potential factors affecting the overall health and all-cause mortality of older adults.

Cognitive impairment is an adverse neurological state defined as the impairment or disruption of one or more key cognitive functions, including learning, attention, memory, or decision-making (2), and is a common issue among older adults. The incidence rates of dementia and mild cognitive impairment (MCI) among Chinese adults 60 years of age or older were estimated to be 6 and 15.5%, respectively (3). Over 50% of patients with MCI progressed to a diagnosis of dementia within 5 years (4). Previous studies have shown that cognitive impairment can lead to disability (5) and an increased risk of mortality (4). Vitamin D deficiency is also a significant concern among older adults, affecting over 30% of Chinese over the age of 65 (6). Vitamin D has been identified as a critical nutrient involved in the regulation of immune function (7), bone health (8), and brain health (9). Deficiencies in vitamin D have been associated with cognitive impairment (10) and increased mortality risk (11) among older adults. Thus, the combination of vitamin D concentration and cognitive status may be useful for the identification of individuals who are at the highest risk of mortality and to determine which factor contributes most significantly to all-cause mortality risk when the other factor is normal. However, while cognitive function and vitamin D have been individually associated with mortality risk, the combined effect of these factors on mortality has not been well-studied. Given the potential interplay between cognitive function and vitamin D on health outcomes, investigating the combined effect of these two factors on mortality is of clinical relevance.

This prospective cohort study aimed to investigate the combined association of cognitive function and vitamin D concentration with all-cause mortality among older adults. We hypothesized that lower cognitive function and vitamin D concentration would be associated with a higher risk of all-cause mortality and that the effect of one factor on mortality may be modified by the other factor. The study findings may provide insight into the formulation of effective interventions to improve health outcomes and the exploration of the mechanisms underlying the associations in older adults. The combination of these two factors influencing all-cause mortality may go beyond a simple additive effect.

## Methods

### Study participants

The data used in this study were derived from the ongoing, prospective, multidisciplinary Chinese Longitudinal Healthy Longevity Survey (CLHLS), which was established to explore health and longevity-related factors among older Chinese individuals (12, 13). Baseline CLHLS surveys were performed in 1998, with follow-ups conducted every 2, 3, or 4 years thereafter. Biomarker-based studies were performed every 3–4 years since 2008 in eight longevity regions.

Participants in the current study were recruited from the 2011–2012 CLHLS biomarker sub-study waves, where follow-ups were conducted in the 2014 and 2018 waves. Individuals eligible for inclusion in this study were adults ≥65 years of age for whom baseline biomarker and cognitive assessment data were available. Participants who were lost to follow-up were excluded. In total, 1,673 participants for whom data were available were included in the analysis to evaluate the effects of vitamin D concentration and cognitive impairment on all-cause mortality. A participant inclusion flowchart is shown in Supplementary Figure S1.

### Outcomes

Participant mortality was recorded during the CLHLS 2014 and 2018 follow-up waves. The date of death was determined according to death certificates or reports from next of kin or neighborhood committees, with survival calculated as the interval between the initial interview and the date of death. Surviving participants at the most recent follow-up were treated as censored observations, with censoring time measured as the interval from the first interview to the last follow-up.

### Cognitive function analyses

The Chinese version of the Mini-Mental State Examination (MMSE), which is the Chinese-adapted version of the international MMSE survey (Supplementary Table S1), was used to assess participants’ cognitive function in this study. The validity and reliability of the Chinese version of this survey have been documented previously (4, 14). The MMSE is composed of six dimensions (time/place orientation, word registration, attention and calculation, memory, visual construction, and language). The total possible score is between 0 and 30. MMSE score thresholds for cognitive impairment were adjusted for level of education such that cognitively impaired individuals were those meeting the following criteria: (1) illiterate individuals with an MMSE score < 18; (2) individuals with 1–6 years of education with MMSE scores <21; (3) individuals with >6 years of education with an MMSE score < 25 (15).

### Vitamin D concentration

25-hydroxyvitamin D [25(OH)D] is the most reliable surrogate measure for vitamin D concentration in the human body (16). Blood samples were collected in heparin-containing vacuum tubes by trained medical professionals and centrifuged at 2500 rpm for 10 min at 20°C. Plasma 25(OH)D concentration was then measured by ELISA (Immunodiagnostic Systems Limited, Bolton, UK). Participants were separated into four groups based on the quartiles of 25(OH)D concentrations (<27.1, 27.1–37.9, 37.9–52.4, and ≥ 52.4 nmol/L). Stratified analysis was performed according to the dichotomous cutoff point of 25(OH) D deficiency as recommended by the Endocrine Society Guidelines: normal vitamin D concentration (≥50 nmol/L) and low vitamin D concentration (<50 nmol/L) (17).

### Covariates

Covariates with the potential to influence study outcomes that were assessed in this analysis included baseline age, sex, marital status [married or other (never married, divorced, or widowed)], residence (urban area or rural area), [living with a family member(s) or others (living alone or in an institution)] drinker status (current, former, or never), smoker status (current, former, or never), regular exercise (current, former, or never), body mass index (BMI), sleep time (<6, 6 to 9, or ≥ 9 h), the season during which blood was collected, chronic comorbidities, albumin concentration, creatinine concentration, and hemoglobin concentration. BMI was calculated using weight (kg)/height (m^2^). BMI < 18.5 kg/m^2^ was defined as underweight, BMI 18.5–24 kg/m^2^ as normal, BMI 24–28 kg/m^2^ as overweight, and BMI ≥28 kg/m^2^ as obese. The chronic comorbidities considered in this study included physician-diagnosed diabetes, stroke, hypertension, cerebrovascular disease, cancer, asthma, pneumonia, emphysema, tuberculosis, and bronchitis, with participants separated into those with 0, 1, 2, or 3+ chronic diseases. The estimated glomerular filtration rate (eGFR) was calculated with the improved 4-variable Chronic Kidney Disease Epidemiology Collaboration (CKD-EPI) formula (adjustment factor 1.1) (18). The central clinical laboratory of Capital Medical University measured all blood biomarker concentrations.

### Data analysis

To compensate for missing data, we adopted a multiple imputation method based on the chain equation method and the five repetitions method (Supplementary Table S2). Continuous variables in this study exhibited skewed distributions. Categorical and baseline continuous variables were compared using chi-square and Kruskal-Wallis tests, respectively. Baseline participant characteristics were reported as medians with interquartile ranges (IQRs) or percentages.

Logistic regression was used to assess the association between vitamin D and cognitive impairment at baseline, with odds ratios (ORs) and 95% confidence intervals (CIs) calculated. Cox proportional hazards models were employed to evaluate the relationship between vitamin D and cognitive status and all-cause mortality, and hazard ratios (HRs) and 95% CIs were calculated. To observe the combined effects of vitamin D and cognitive status on all-cause mortality, we categorized 25(OH)D and cognitive status into binary variables and created a 4-level joint 25(OH)D and cognition variable as follows: Group 1: 25(OH)D ≥ 50 nmol/L and normal cognition; Group 2: 25(OH)D < 50 nmol/L and normal cognition; Group 3: 25(OH)D ≥ 50 nmol/L and cognitive impairment; Group 4: 25(OH)D < 50 nmol/L and cognitive impairment. Schoenfeld residuals were additionally utilized to test the proportionality assumption, with the result being met (*p* > 0.05 for all-cause mortality). Based on Kaplan–Meier and log-rank tests, survival curves were estimated. Crude incidence rates per 100 person-years of all-cause mortality were estimated. Additionally, we analyzed the dose–response relationship between 25(OH)D concentration and cognitive impairment as well as the risk of all-cause mortality using restricted cubic spline analysis. The analysis included five knots at the 5.0th, 27.5th, 50.0th, 72.5th, and 95.0th percentiles of 25(OH)D, with a reference set at 50 nmol/L. Candidate variables with *p*-values <0.05 on the univariate analysis for cognitive function were included as covariates in adjusted models. We set up the unadjusted model, age and sex-adjusted model, and the multivariable-adjusted model for the outcome. Multiplicative interactions were examined by incorporating a cross-product term corresponding to cognitive function and plasma 25(OH)D to examine whether the association of 25(OH)D concentration with the risk of all-cause mortality was modified by cognitive status. To examine the robustness of our findings, we performed the sensitivity analysis by analyzing complete cases to evaluate the potential effects of the multiple imputation method.

All analyses were performed using R v 4.1.3 (R Foundation for Statistical Computing), with a two-tailed *p* < 0.05 as the significance threshold.

## Results

Of the 1,673 individuals enrolled in the present study cohort, 420 (25.1%) exhibited baseline cognitive impairment. The characteristics of participants stratified according to cognitive function status are shown in Table 1. Relative to participants exhibiting normal cognitive function, those suffering from cognitive impairment had a higher chance of being female, older, not married (unmarried, divorced, or widowed), non-drinkers, non-smokers, lacking exercise and living with family members, as well as having long sleep times and lower BMI. These cognitively impaired participants were also more likely to exhibit low albumin, eGFR, hemoglobin, and as well as 25(OH)D concentration.

**Table 1:** Baseline participant characteristics.

Characteristics	Overall (*N* = 1,673)	Normal cognition (*N* = 1,253)	Cognitive impairment (*N* = 420)	*p* value^a^
Age (years), median (IQR)	87.00 (76.00, 99.00)	82.00 (73.00, 92.00)	100.00 (94.00, 102.00)	<0.001
Female, no. (%)	930 (55.6)	603 (48.1)	327 (77.9)	<0.001
Married, no. (%)	631 (37.7)	594 (47.4)	37 (8.8)	<0.001
Rural area, no. (%)	1,404 (83.9)	1,044 (83.3)	360 (85.7)	0.28
Living with a family member(s), no. (%)	1,293 (77.3)	952 (76.0)	341 (81.2)	0.03
Smoking status, no. (%)				<0.001
Current	271 (16.2)	241 (19.2)	30 (7.1)	
Former	138 (8.2)	117 (9.3)	21 (5.0)	
Never	1,264 (75.6)	895 (71.4)	369 (87.9)	
Drinking status, no. (%)				<0.001
Current	256 (15.3)	219 (17.5)	37 (8.8)	
Former	104 (6.2)	86 (6.9)	18 (4.3)	
Never	1,313 (78.5)	948 (75.7)	365 (86.9)	
Regular exercise, no. (%)				<0.001
Current	232 (13.9)	206 (16.4)	26 (6.2)	
Former	43 (2.6)	30 (2.4)	13 (3.1)	
Never	1,398 (83.6)	1,017 (81.2)	381 (90.7)	
BMI (kg/m^2^), no. (%)				<0.001
<18.5	448 (26.8)	284 (22.7)	164 (39.0)	
18.5–24	895 (53.5)	684 (54.6)	211 (50.2)	
24–28	253 (15.1)	222 (17.7)	31 (7.4)	
≥28	77 (4.6)	63 (5.0)	14 (3.3)	
Total sleep time (h), no. (%)				<0.001
<6	293 (17.5)	215 (17.2)	78 (18.6)	
6–9	1,015 (60.7)	806 (64.3)	209 (49.8)	
>9	365 (21.8)	232 (18.5)	133 (31.7)	
Chronic disease, no. (%)				0.12
None	1,025 (61.3)	747 (59.6)	278 (66.2)	
One chronic disease	470 (28.1)	366 (29.2)	104 (24.8)	
Two chronic diseases	143 (8.5)	113 (9.0)	30 (7.1)	
Three or more chronic diseases	35 (2.1)	27 (2.2)	8 (1.9)	
Annual household income (yuan), no. (%)				0.25
<10,000	709 (42.4)	540 (43.1)	169 (40.2)	
10,000–30,000	566 (33.8)	410 (32.7)	156 (37.1)	
>30,000	398 (23.8)	303 (24.2)	95 (22.6)	
Season of blood draw, no. (%)				<0.001
Spring (March–May)	451 (27.0)	313 (25.0)	138 (32.9)	
Summer (June–August)	1,142 (68.3)	869 (69.4)	273 (65.0)	
Autumn (September–November)	80 (4.8)	71 (5.7)	9 (2.1)	
25(OH) D (nmol/L), median (IQR)	36.90 (26.83, 51.46)	40.86 (29.80, 55.50)	27.60 (20.44, 37.31)	<0.001
Albumin (g/L), median (IQR)	40.10 (36.90, 43.30)	40.80 (37.90, 43.90)	37.70 (35.10, 40.70)	<0.001
eGFR (ml/min per 1.73 m^2^), no. (%)				<0.001
≥90	382 (22.8)	352 (28.1)	30 (7.1)	
60–90	897 (53.6)	636 (50.8)	261 (62.1)	
30–60	359 (21.5)	242 (19.3)	117 (27.9)	
15–30	31 (1.9)	21 (1.7)	10 (2.4)	
<15	4 (0.2)	2 (0.2)	2 (0.5)	
Hemoglobin (g/L), median (IQR)	123.00 (110.00, 137.00)	125.00 (112.00, 140.00)	118.00 (105.00, 132.00)	<0.001

^a^*p*-values for continuous and categorical variables were, respectively, calculated using Kruskal-Wallis and chi-square tests. BMI, body mass index; 25(OH)D, 25-Hydroxyvitamin D; eGFR, estimated glomerular filtration rate; IQR, interquartile range.

We compared the baseline characteristics of participants who were followed up successfully with those who were lost to follow-up (Supplementary Table S3). We found that participants who were lost to follow-up were more likely to be younger, former smokers and drinkers, married, have regular exercise, have higher annual household income, and have lower hemoglobin and higher plasma 25(OH)D concentration.

### Associations between 25(OH)D and cognitive impairment

After adjusting for potential confounders, individuals with low vitamin D concentrations (<50 nmol/L) exhibited a higher risk of cognitive impairment compared to those with normal concentrations (≥50 nmol/L) (OR 3.17, 95% CI: 2.06 to 5.00) (Supplementary Figure S2). Furthermore, the OR for cognitive impairment was 5.53 (95% CI: 3.34 to 9.44) among participants in the lowest quartile of plasma 25(OH)D concentration (<27.1 nmol/L) compared to those in the highest quartile (≥52.4 nmol/L) (Supplementary Figure S2). The association between 25(OH)D and cognitive impairment was a linear trend (inverted J-shaped) (*p* for nonlinearity = 0.07); low 25(OH)D concentrations were associated with cognitive impairment (Supplementary Figure S3).

### Individual associations of 25(OH)D and cognitive status with all-cause mortality

**Figure 1:**
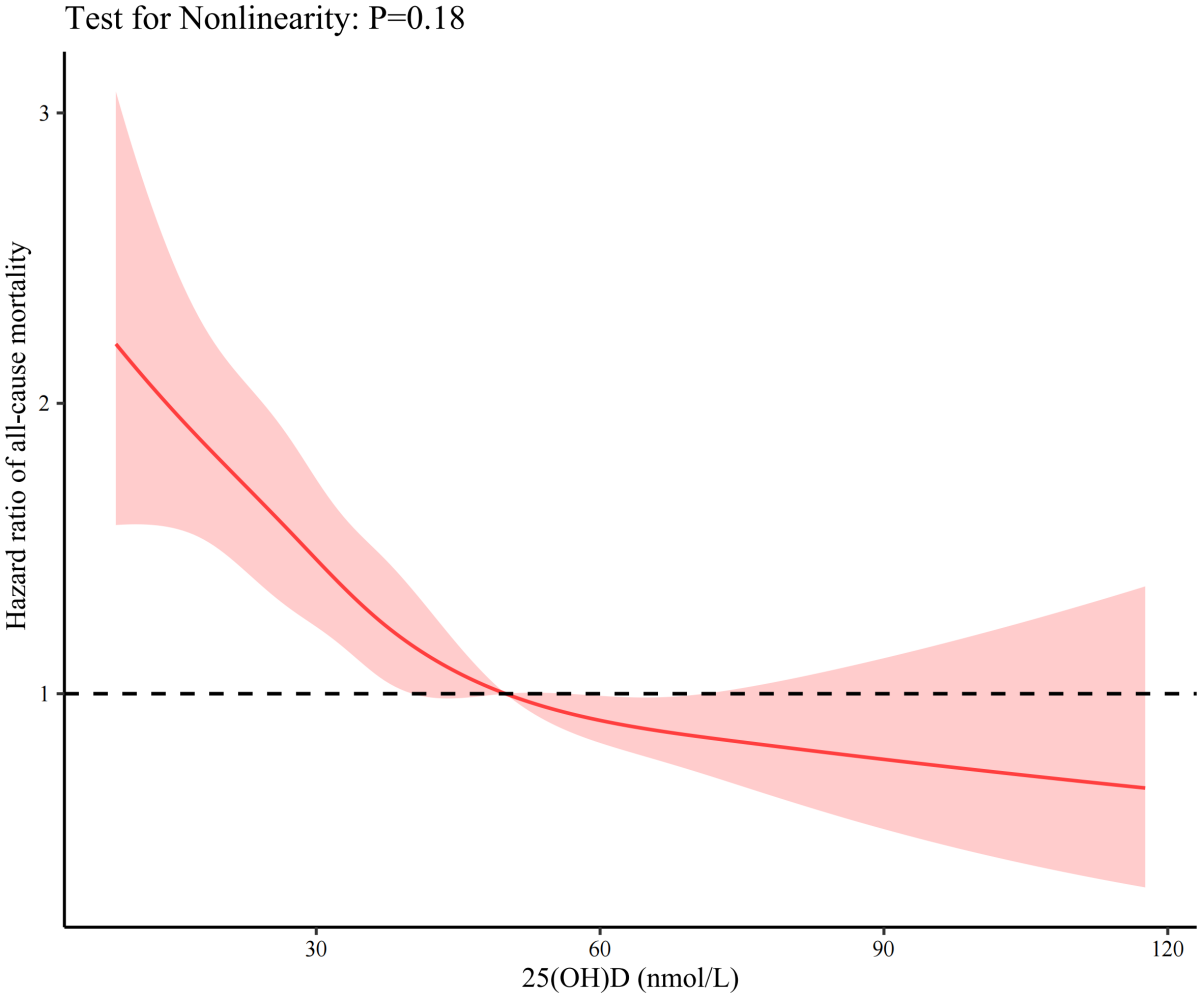
Dose–response association between vitamin D and risk of all-cause mortality. Hazard ratios are indicated by solid red lines and 95% CIs by shaded areas. The model was adjusted for age, sex, the season of blood draw, living arrangement, marital status, drinking status, smoking status, regularity of exercise, body mass index, cognitive status, estimated glomerular filtration rate, hemoglobin, and albumin concentration.

Over a total of 6292.8 person-years of follow-up, 899 (53.7%) participants died, with a mean ± SD follow-up period of 3.8 ± 1.9 years. Based on the Kaplan–Meier curves illustrating survival probability, it was found that participants belonging to the lowest quartile of 25(OH)D concentration at baseline exhibited the lowest survival probabilities as compared to those belonging to the other quartiles (as presented in Supplementary Figure S4). Table 2 shows that participants with the lowest quartile of 25(OH) D were 2.11 times more likely to die than those with the highest quartile of 25(OH) D in the final model (*p* for trend < 0.05). Similar statistically significant results were obtained when 25(OH)D concentrations were classified using the cutoff value of 50 nmol/L. Figure 1 shows the smoothed spline curve of the HRs for 25(OH)D as continuous variables. The fully adjusted smooth curve showed a linear association between 25(OH)D and all-cause mortality (*p* for nonlinearity = 0.182). There was a steep increase in the odds of death with 25(OH)D concentration <39.98 nmol/L. Similarly, cognitive impairment was associated with a higher risk of all-cause mortality (HR 1.81, 95% CI: 1.54 to 2.12) (Table 2).

**Table 2:** Hazard ratios for the individual associations of vitamin D concentration and cognitive status with all-cause mortality (*N* = 1,673).

Characteristics	Mortality, no. (per 100 person-year, %)	Unadjusted model	Model 1	Model 2
		HR (95% CI)	HR (95% CI)	HR (95% CI)
25(OH)D (nmol/L)				
Cut-off at 50 nmol/L				
Low vitamin D (<50)	731 (17.2)	2.11 (1.78, 2.50) ^***^	1.69 (1.42, 2.01) ^***^	1.63 (1.35, 1.98) ^***^
Normal vitamin D (≥50)	168 (8.2)	Reference	Reference	Reference
Cut-offs by quartiles				
Quartile 1 (<27.1)	312 (25.4)	3.14 (2.59, 3.82) ^***^	2.05 (1.68, 2.51) ^***^	2.11 (1.68, 2.66) ^***^
Quartile 2 (27.1–37.9)	238 (15.7)	1.92 (1.57, 2.36) ^***^	1.55 (1.26, 1.91) ^***^	1.53 (1.22, 1.92) ^***^
Quartile 3 (37.9–52.4)	197 (11.7)	1.44 (1.16, 1.78) ^***^	1.40 (1.14, 1.73) ^**^	1.36 (1.09, 1.71) ^**^
Quartile 4 (≥52.4)	152 (8.2)	Reference	Reference	Reference
*p* for trend		<0.001	<0.001	<0.001
Cognitive function				
Cognitive impairment	379 (40.0)	4.34 (3.79, 4.97) ^***^	1.90 (1.63, 2.22) ^***^	1.81 (1.54, 2.12) ^***^
Normal cognition	520 (9.7)	Reference	Reference	Reference

Model 1 adjusted for age and sex; model 2 further adjusted for the season of blood draw, living arrangement, marital status, drinking status, smoking status, regularity of exercise, body mass index, estimated glomerular filtration rate, hemoglobin, and albumin concentration, and further adjusted for cognitive status in vitamin D model, and 25(OH)D concentrations in cognitive function model. ^**^p < 0.01, ^***^p < 0.001. 25(OH) D, 25-Hydroxyvitamin D; HR, hazard ratio; CI, confidence interval.

### Combined associations of 25(OH)D and cognitive status on all-cause mortality

**Figure 2:**
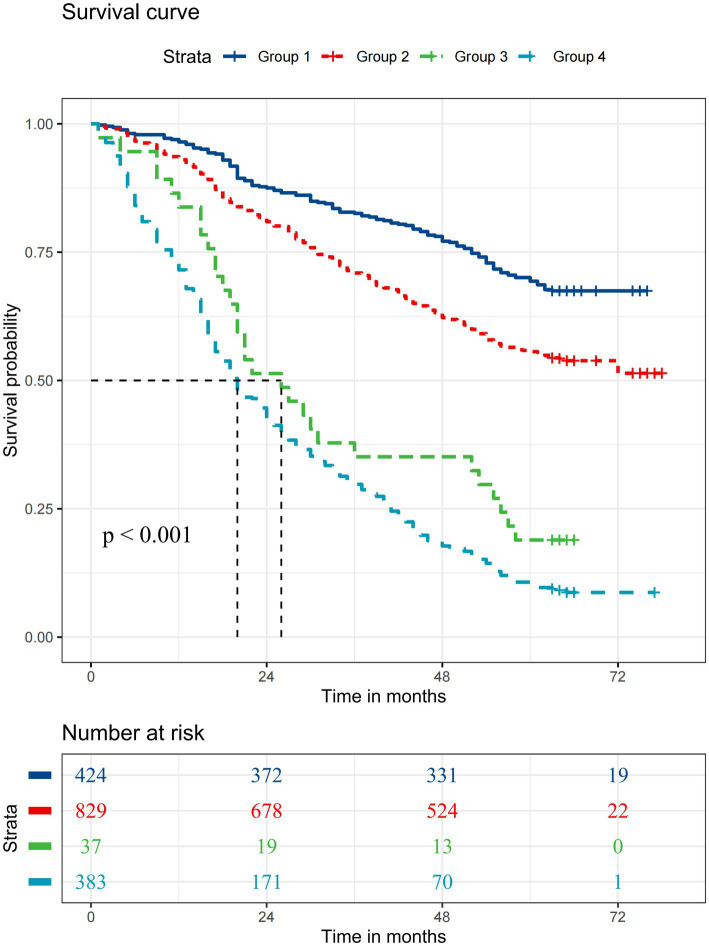
Kaplan–Meier survival curves for all-cause mortality according to 25-Hydroxyvitamin D and cognition function. The median survival duration is represented by a vertical dashed line. Group 1: 25-Hydroxyvitamin D ≥ 50 nmol/L and normal cognition; Group 2: 25-Hydroxyvitamin D < 50 nmol/L and normal cognition; Group 3: 25-Hydroxyvitamin D ≥ 50 nmol/L and cognitive impairment; Group 4: 25-Hydroxyvitamin D < 50 nmol/L and cognitive impairment.

**Figure 3:**
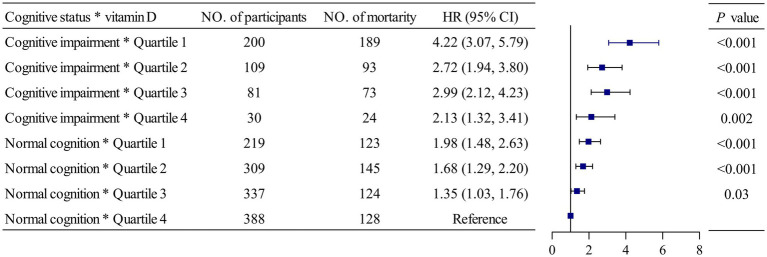
The interactive effect of vitamin D and cognitive function on all-cause mortality. The model was adjusted for age, sex, the season of blood draw, living arrangement, marital status, drinking status, smoking status, regularity of exercise, body mass index, estimated glomerular filtration rate, hemoglobin, and albumin concentration. HR, hazard ratio, CI, confidence interval.

Results from Kaplan–Meier analyses of 25(OH) D and cognitive function, once binarized, are reported in Figure 2. Participants with low vitamin D concentration and cognitive impairment had the lowest survival probabilities compared to those in the remaining groups. Supplementary Figure S5 portrays the Kaplan–Meier survival curves based on 25(OH)D quartiles and cognitive function. The most reduced survival rates were observed in those participants with 25(OH)D quartile 1 and cognitive impairment, and the highest survival rates were seen in participants with 25(OH)D quartile 4 and normal cognitive function. Positive associations were observed in the combined analysis, where it was found that the highest risk of mortality was associated with both low vitamin D concentration and cognitive impairment (group 4) (HR 3.04, 95% CI: 2.40 to 3.86) (Table 3). When assessing the risk of all-cause mortality, a significant interaction was detected between vitamin D concentration and cognitive function (*p* for interaction <0.001) (Figure 3).

**Table 3:** Hazard ratios for the combined associations of vitamin D concentration and cognitive impairment with all-cause mortality (*N* = 1,673).

Groups	Mortality, no. (per 100 person-year, %)	Unadjusted model	Model 1	Model 2
		HR (95% CI)	HR (95% CI)	HR (95% CI)
Low vitamin D and cognitive impairment	349 (41.3)	6.23 (5.10, 7.61) ^***^	2.70 (2.17, 3.36) ^***^	3.04 (2.40, 3.86) ^***^
Normal vitamin D and cognitive impairment	30 (29.3)	4.27 (2.88, 6.35) ^***^	1.63 (1.09, 2.45) ^*^	1.94 (1.29, 2.93) ^**^
Low vitamin D and normal cognition	382 (11.3)	1.60 (1.32, 1.95) ^***^	1.51 (1.24, 1.84) ^***^	1.65 (1.33, 2.04) ^***^
Normal vitamin D and normal cognition	138 (7.1)	Reference	Reference	Reference

Model 1 adjusted for age and sex; model 2 further adjusted for the season of blood draw, living arrangement, marital status, drinking status, smoking status, regularity of exercise, body mass index, estimated glomerular filtration rate, hemoglobin, and albumin concentration. ^*^p < 0.05, ^**^p < 0.01, ^***^p < 0.001. HR, hazard ratio; CI, confidence interval.

### Sensitivity analysis

Sensitivity analysis indicated no change in the association between 25(OH)D, cognitive status, and all-cause mortality (Supplementary Tables S4–S6).

## Discussion

In the present prospective analysis of 1,673 Chinese adults over the age of 65 years, both cognitive impairment and lower vitamin D concentrations were independently associated with an elevated risk of all-cause mortality. Furthermore, the combined presence of vitamin D deficiency and cognitive impairment was associated with a higher risk of all-cause mortality. The significant interaction between cognitive function and vitamin D indicates that the effect of one factor on mortality may be modified by the other factor.

The study’s findings are consistent with previous research that has shown that cognitive function and vitamin D concentration are individually significant predictors of mortality risk. One study of 77,541 individuals 65 years of age or older found that relative to normal cognition, mild, moderate, and severe cognitive impairment were significantly associated with mortality risk, with respective HRs of 1.67 (95% CI: 1.43–1.94), 2.26 (95% CI: 1.90–2.70), and 2.68 (95% CI: 2.25–3.19) (19). A separate prospective large-scale study found that cognitive impairment, measured using education level-adjusted MMSE scores, was closely related to all-cause mortality risk among adults 65 years of age or older (20). Similarly, previous studies have shown that low concentrations of vitamin D are linked to an increased risk of mortality (21, 22). Studies exploring the prognostic implications of the combined effects of vitamin D concentration and cognitive impairment, however, have been limited to date.

The results of this study also suggest that vitamin D and cognitive function may act synergistically on mortality risk. The mechanisms underlying the association between cognitive function and vitamin D and mortality are not well understood. It has been suggested that cognitive function has been linked to various physiological and psychological factors, including inflammation (23), oxidative stress (24), and depression (25), which may contribute to all-cause mortality. In addition, cognitive impairment may lead to poor health-related behaviors, such as a sedentary lifestyle (26), poor nutrition (27), and poor adherence to medical treatments (28), which may also increase the risk of mortality. Vitamin D is involved in several physiological processes, including immune regulation, anti-inflammatory activity (7), and calcium metabolism (8), and it has been suggested that a low concentration of vitamin D may lead to increased inflammation and disordered calcium metabolism, increasing the risk of mortality (29). Thus, we speculate that the combined associations of cognitive function and vitamin D with all-cause mortality may reflect the cumulative effects of multiple pathophysiological processes. Furthermore, our study found that participants with normal cognitive function were more likely to have high concentrations of albumin and 25(OH)D. This may support previous research which indicates that following a diet rich in lean protein, soy-derived products, fruits, and vegetables can have a protective effect against cognitive impairment (30), and certain vitamins present in these foods, such as vitamin D, folic acid, and B vitamins, may also contribute to cognitive function (31). Prior studies have demonstrated that vitamin D has neuroprotective properties, including regulating neuronal calcium levels, modulating neurotransmitter synthesis, and promoting neurotrophic factor expression (32, 33). Our study reinforces earlier research that points to a beneficial relationship between vitamin D and cognitive function in older adults. While it is known that unhealthy dietary habits can impact cognitive function, including potential effects from inadequate intake of vitamin D and decreased albumin concentration (34, 35), unfortunately, our study did not further explore a definitive relationship between these factors. Similarly, cognitively impaired adults may be more likely to develop a vitamin D deficiency due to lower levels of physical activity (36, 37). As such, it also may be that vitamin D and cognitive function interact and work together to increase the risk of all-cause mortality.

According to the results of our study, the combined effect of lower concentrations of vitamin D and cognitive impairment on mortality surpasses the simple additive effect of each factor individually. This intriguing finding has significant implications for the development of health interventions targeting this population. Rather than solely targeting individual risk factors such as vitamin D or cognitive impairment, a comprehensive approach that addresses both factors may be more effective in reducing mortality rates. This approach could involve multidimensional interventions, including modifications to lifestyle and diet, as well as supplementation with vitamin D and cognitive training programs. To gain a better understanding of how these factors interact to affect mortality, further research is needed. It would be beneficial to investigate how varying concentrations of vitamin D and cognitive function interact with one another and to identify other variables that may be related to all-cause mortality in older adults with low concentrations of vitamin D and cognitive impairment. Overall, our study provides crucial insights into the heightened risk associated with low concentrations of vitamin D and cognitive impairment in older adults. These findings can be utilized to inform the development of targeted health interventions aimed at reducing all-cause mortality among this population.

### Strengths and limitations

A key strength of this study is that the data were derived from a representative population of older Chinese adults, allowing for a longitudinal analysis of the interplay between vitamin D concentration, cognitive impairment, and all-cause mortality over six years. However, there are multiple limitations to this analysis that should be taken into consideration. First, the study did not account for changes in cognitive function or vitamin D concentration over time, which may have affected the associations. Longitudinal measures of cognitive function and 25(OH)D concentration should be incorporated in future studies to mitigate the potential impact of changes in these factors over time on study outcomes. Second, some covariates of the study were based on self-reported data, which may be subject to recall bias. Finally, the results of these analyses may have been impacted by other covariates that were not measured or considered.

## Conclusion

In conclusion, this study provides evidence that both cognitive function and vitamin D are independently associated with all-cause mortality among older adults. The results also suggest that the combination of these two factors may have a synergistic effect on all-cause mortality risk. The significant interaction between cognitive function and vitamin D suggests that interventions targeting both cognitive function and vitamin D concentration may have the potential to reduce the risk of all-cause mortality in older adults. Further research is needed to determine the mechanisms underlying these associations and to explore the potential benefits of interventions targeting cognitive function and vitamin D.

## Data availability statement

The original contributions presented in the study are included in the article/Supplementary material, further inquiries can be directed to the corresponding author.

## Ethics statement

The studies involving human participants were reviewed and approved by The Biomedical Ethics Committee of Peking University approved this study (IRB00001052-13074). The patients/participants provided their written informed consent to participate in this study.

## Author contributions

NG and MD conceptualized and designed the study. MD, QS, XW, TL, RL, TJ and XS organized the database, analyzed data, and prepared and reviewed figures. MD and QS wrote the original draft. NG, YL, and JY provided critical revisions of the manuscript. All authors read and approved the submitted version.

## Funding

This study was supported by grants from the National Key Research and Development Program of China (2020YFC2005300), Major Science & Technology Program of Sichuan Province (2022ZDZX0021), 1·3·5 project for disciplines of excellence-Clinical Research Incubation Project, West China Hospital, Sichuan University (19HXFH012), National Clinical Research Center for Geriatrics, West China Hospital, Sichuan University (Z20191003), and National Clinical Research Center for Geriatrics, West China Hospital, Sichuan University (Z20191012). We thank the CLHLS who collected and reported data and made the data publicly available to researchers. The funder had no role in the study design, data collection, data analysis, or preparation of this manuscript.

## Conflict of interest

The authors declare that the research was conducted in the absence of any commercial or financial relationships that could be construed as a potential conflict of interest.

## Publisher’s note

All claims expressed in this article are solely those of the authors and do not necessarily represent those of their affiliated organizations, or those of the publisher, the editors and the reviewers. Any product that may be evaluated in this article, or claim that may be made by its manufacturer, is not guaranteed or endorsed by the publisher.
