# Supplementary material for: Combined associations of vitamin D and cognitive function with all-cause mortality among older adults in Chinese longevity areas: A prospective cohort study
Source: Front Public Health. 2023 May 2;11:1024341. doi: 10.3389/fpubh.2023.1024341 (PMC10189877; doi:10.3389/fpubh.2023.1024341)
Supplement: Supplementary file 1 [file Table_1.pdf]

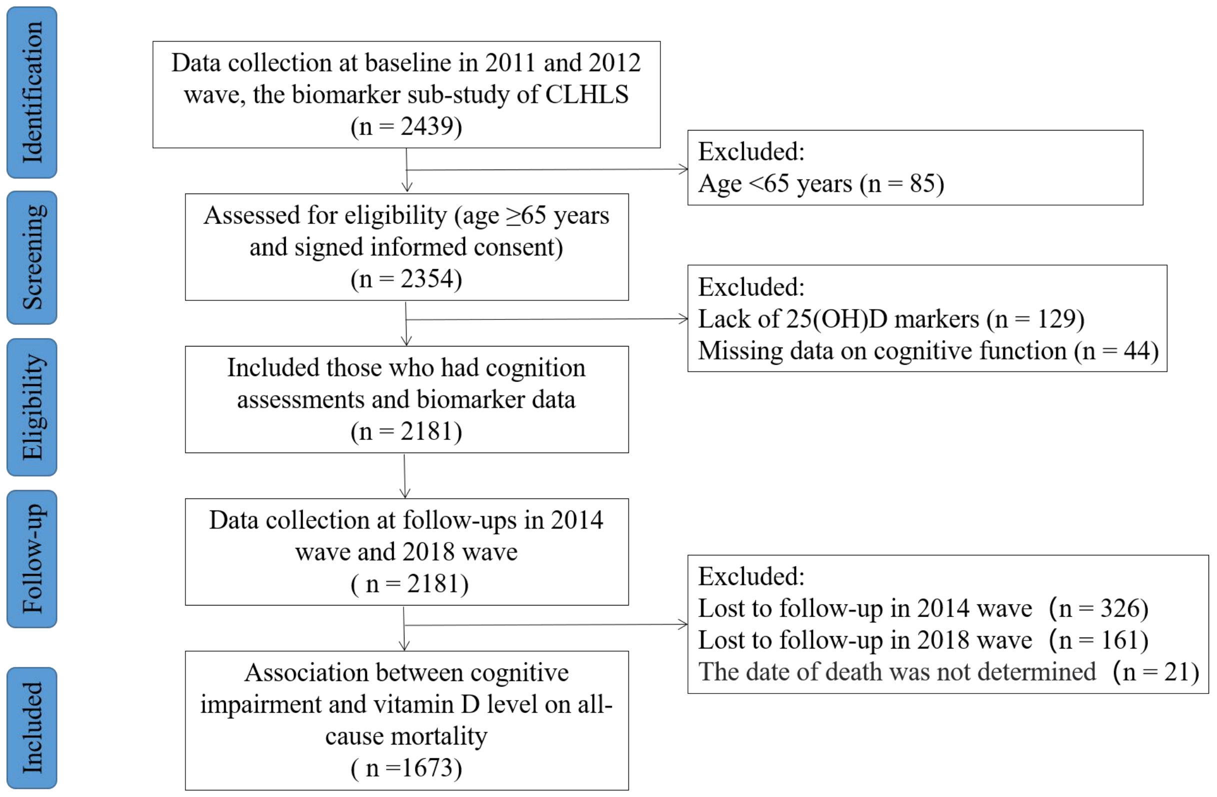

Figure S1 Flowchart of the included study population. *CLHLS*, Chinese Longitudinal Healthy Longevity Survey.

25(OH)D (nmol/L)	NO. of participants	NO. of cognitive impairment	OR (95% CI)	*P* value
Cut-off at 50 nmol/L				
Low vitamin D (<50)	1212	383	3.17 (2.06, 5.00)	<0.001
Normal vitamin D (≥50)	461	37	Reference	
Cut-offs by quartiles				
Quartile 1 (<27.1)	419	200	5.53 (3.34, 9.44)	<0.001
Quartile 2 (27.1-37.9)	418	109	2.98 (1.75, 5.20)	<0.001
Quartile 3 (37.9-52.4)	418	81	2.84 (1.69, 4.89)	<0.001
Quartile 4 (≥52.4)	418	30	Reference	

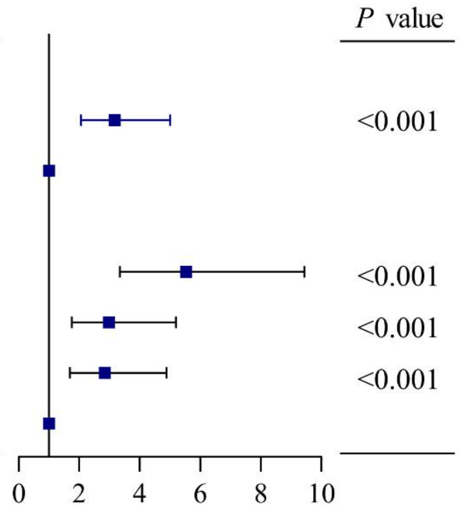

Figure S2 The association between vitamin D concentrations and cognition impairment. The model was adjusted for age, sex, the season of blood draw, living arrangement, marital status, drinking status, smoking status, regularity of exercise, body mass index, estimated glomerular filtration rate, hemoglobin, and albumin concentration. *25(OH)D*, 25-Hydroxyvitamin D; *OR,* odd ratio; *CI,* confidence interval.

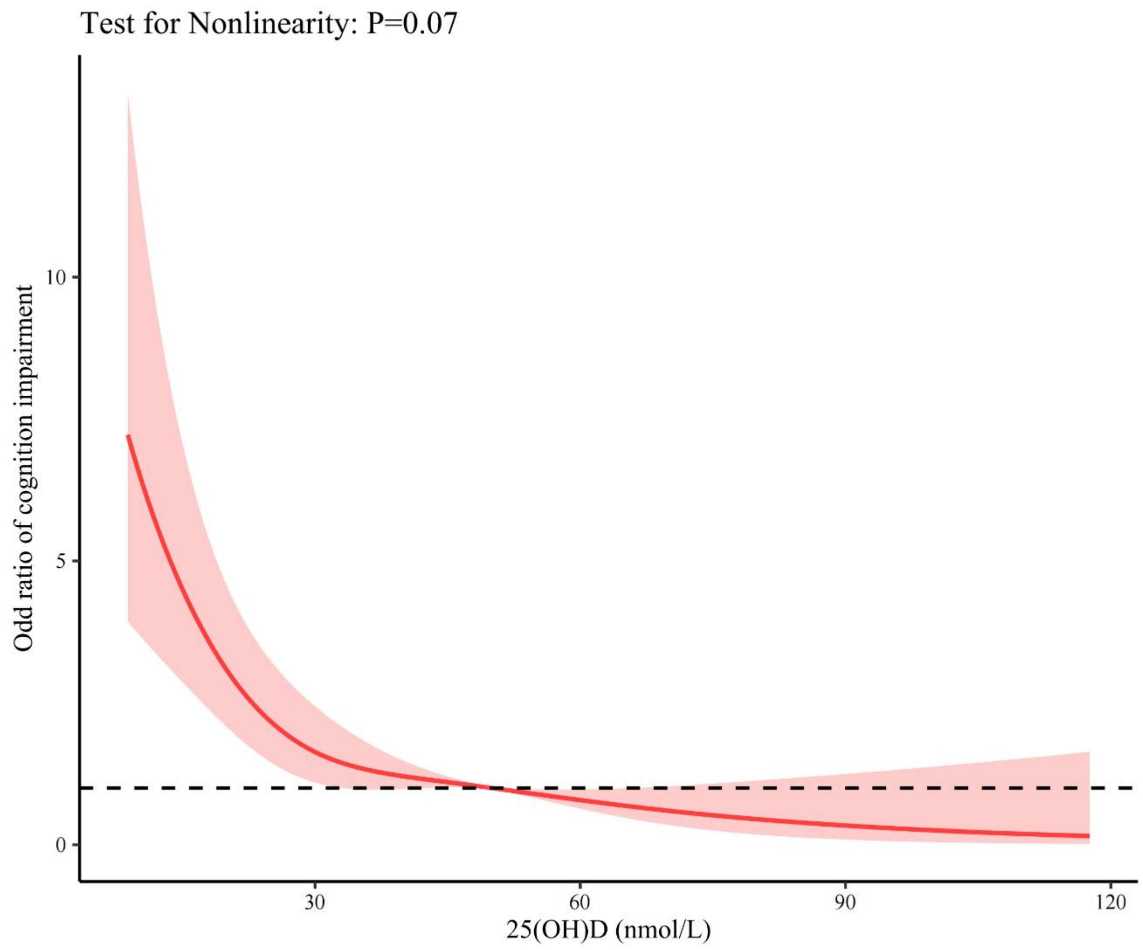

Figure S3 Dose-response association between vitamin D and cognitive impairment. Odd ratios are indicated by solid red lines and 95% CIs by shaded areas. The model was adjusted for age, sex, the season of blood draw, living arrangement, marital status, drinking status, smoking status, regularity of exercise, body mass index, estimated glomerular filtration rate, hemoglobin, and albumin concentration.

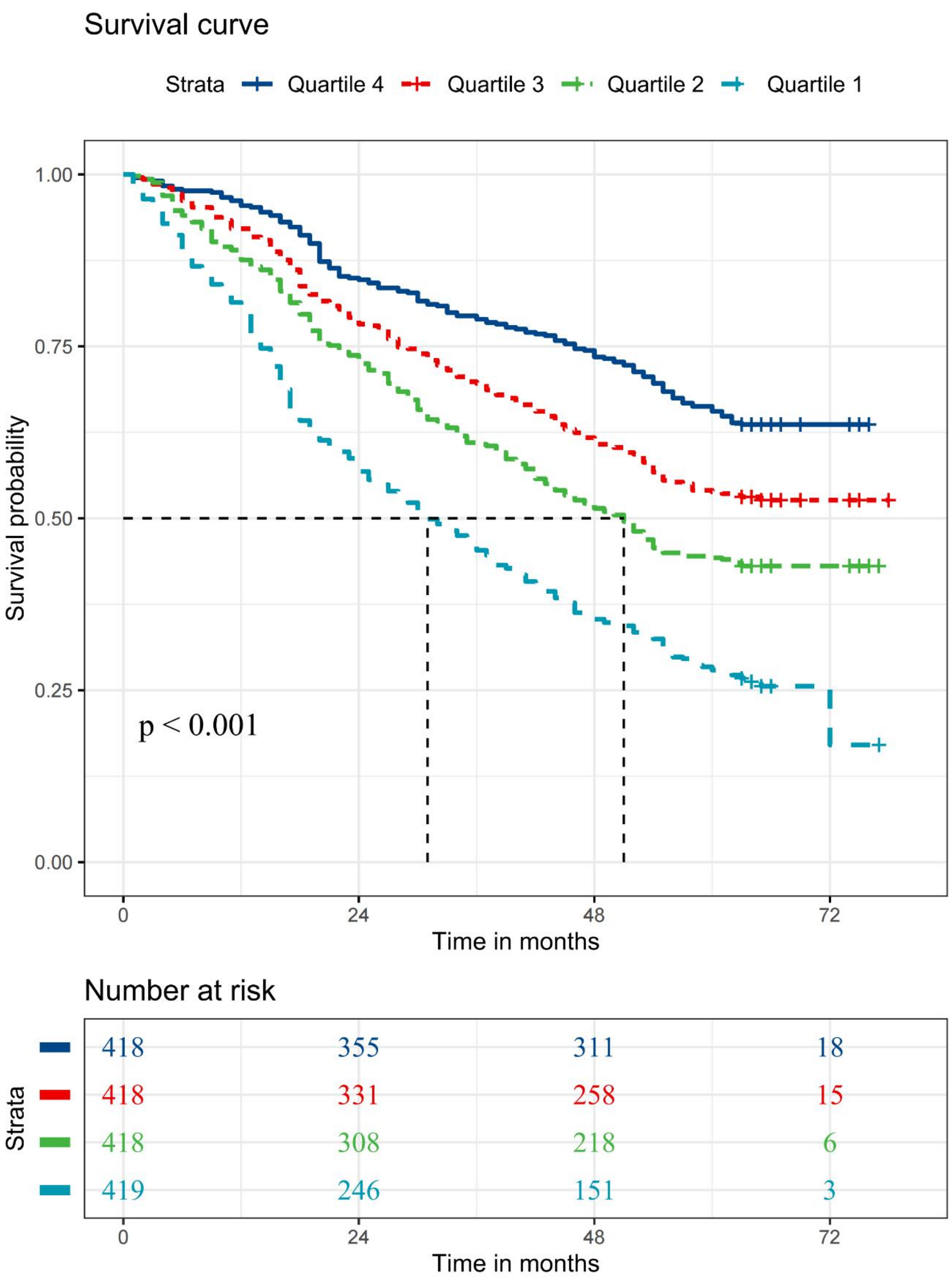

Figure S4 Kaplan-Meier survival curves for all-cause mortality according to 25-Hydroxyvitamin D quartiles. The median survival duration is represented by a vertical dashed line.

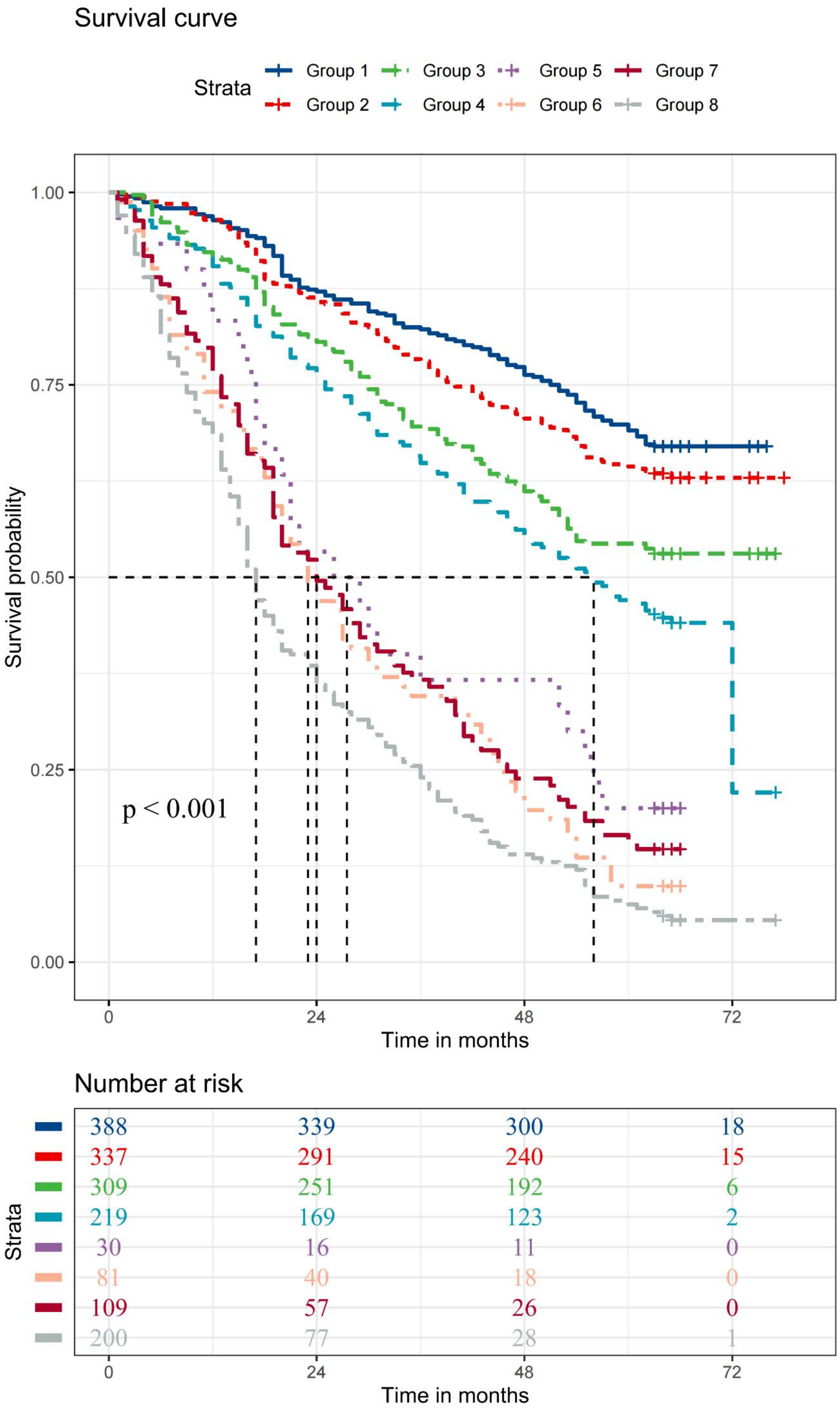

Figure S5 Kaplan-Meier survival curves for all-cause mortality according to 25-Hydroxyvitamin D quartiles and cognition function. The median survival duration is represented by a vertical dashed line. Group 1: The fourth quartile of 25-hydroxyvitamin D and normal cognition. Group 2: The third quartile of 25-hydroxyvitamin D and normal cognition; Group 3: The second quartile of 25-hydroxyvitamin D and normal cognition; Group 4: The first quartile of 25-hydroxyvitamin D and normal cognition; Group 5: The fourth quartile of 25-hydroxyvitamin D and cognitive impairment; Group 6: The third quartile of 25-hydroxyvitamin D and cognitive impairment; Group 7: The second quartile of 25-hydroxyvitamin D and cognitive impairment; Group 8: The first quartile of 25-hydroxyvitamin D and cognitive impairment;

Table S1

The Chinese Version of the Mini-Mental State Exam (MMSE).

Item	MMSE	Score (Total =30)
1. Orientation	What time of day is it right now (morning, afternoon, evening)?	1
	What is the animal year of this year?	1
	What is the date (day and month) of the mid-autumn festival?	1
	What is the season right now?	1
	What is the name of this county or district?	1
2. Naming foods	Please name as many kinds of food as possible in 1 minute (1 point for each food and 7 points for those who name 7 or more foods)	7
3. Registration	Table, apple, cloth. Please repeat these three objects.	3
4. Attention and calculation	I will ask you to spend $3 from $20, then you must spend $3 from the number you arrived at and continue to spend $3 until you are asked to stop.	5
5. Copy a figure	The individual is asked to draw a figure of overlapping pentagons.	1
6. Recall	Name the three objects learned earlier (table, apple, and cloth).	3
7. Language	Naming pen and watch	2
	Repeating the following sentence: “What you plant, what you will get.”	1
	The individual is asked to follow the interviewer’s instruction: “Take the paper using your right hand, fold it in the middle using both hands, and place the paper on the floor.”	3

Table S2

Distributions of variables with missing data comparing observed complete case data to results from 5 imputed datasets with imputed variables from multiple imputations.

	Missing data, no. (%)	Complete case	Multiple imputation1	Multiple imputation2	Multiple imputation3	Multiple imputation4	Multiple imputation5
Living arrangement, no. (%)	41 (2.5)	1,130 (77.0)	1,293 (77.3)	1,296 (77.5)	1,295 (77.4)	1,297 (77.5)	1,298 (77.6)
Smoking status, no. (%)	10 (0.6)						
Current		249 (17.0)	271 (16.2)	273 (16.3)	272 (16.3)	275 (16.4)	271 (16.2)
Former		122 (8.3)	138 (8.2)	137 (8.2)	138 (8.2)	137 (8.2)	139 (8.3)
Never		1,097 (74.7)	1,264 (75.6)	1,263 (75.5)	1,263 (75.5)	1,261 (75.4)	1,263 (75.5)
Drinking status, no. (%)	9 (0.5)						
Current		240 (16.3)	256 (15.3)	257 (15.4)	258 (15.4)	255 (15.2)	257 (15.4)
Former		88 (6.0)	104 (6.2)	103 (6.2)	103 (6.2)	105 (6.3)	103 (6.2)
Never		1,140 (77.7)	1,313 (78.5)	1,313 (78.5)	1,312 (78.4)	1,313 (78.5)	1,313 (78.5)
Regular exercise, no. (%)	45 (2.7)						
Current		217 (14.8)	232 (13.9)	229 (13.7)	229 (13.7)	234 (14.0)	234 (14.0)
Former		35 (2.4)	43 (2.6)	42 (2.5)	42 (2.5)	42 (2.5)	44 (2.6)
Never		1,216 (82.8)	1,398 (83.6)	1,402 (83.8)	1,402 (83.8)	1,397 (83.5)	1,395 (83.4)
Annual household income (yuan), no. (%)	72 (4.3)						
<10,000		626 (42.6)	709 (42.4)	712 (42.6)	714 (42.7)	712 (42.6)	708 (42.3)
10,000-30,000		491 (33.4)	566 (33.8)	564 (33.7)	560 (33.5)	564 (33.7)	564 (33.7)
>30,000		351 (23.9)	398 (23.8)	397 (23.7)	399 (23.8)	397 (23.7)	401 (24.0)
Married, no. (%)	9 (0.5)	569 (38.8)	631 (37.7)	630 (37.7)	630 (37.7)	631 (37.7)	632 (37.8)
Total sleep time (h), no. (%)	10 (0.6)						
<6		259 (17.6)	293 (17.5)	294 (17.6)	292 (17.5)	292 (17.5)	291 (17.4)
6-9		900 (61.3)	1,015 (60.7)	1,014 (60.6)	1,015 (60.7)	1,013 (60.5)	1,014 (60.6)
>9		309 (21.0)	365 (21.8)	365 (21.8)	366 (21.9)	368 (22.0)	368 (22.0)
BMI (kg/m^2), median (IQR)	90 (5.4)	20.5 (18.4, 23.4)	20.5 (18.4, 23.3)	20.5 (18.4, 23.4)	20.5 (18.4, 23.4)	20.5 (18.4, 23.4)	20.5 (18.4, 23.4)
eGFR (ml/min per 1.73 m^2), no. (%)	3 (0.2)						
≥90		3 (0.2)	4 (0.2)	4 (0.2)	4 (0.2)	4 (0.2)	4 (0.2)
60-90		27 (1.8)	31 (1.9)	32 (1.9)	31 (1.9)	31 (1.9)	31 (1.9)
30-60		310 (21.1)	359 (21.5)	359 (21.5)	358 (21.4)	359 (21.5)	359 (21.5)
15-30		782 (53.3)	897 (53.6)	896 (53.6)	898 (53.7)	897 (53.6)	897 (53.6)
<15		346 (23.6)	382 (22.8)	382 (22.8)	382 (22.8)	382 (22.8)	382 (22.8)
Albumin(g/L), median (IQR)	3 (0.2)	40.2 (37.2, 43.4)	40.1 (36.9, 43.3)	40.1 (36.9, 43.3)	40.1 (36.9, 43.3)	40.1 (36.9, 43.3)	40.1 (36.9, 43.3)
Hemoglobin(g/L), median (IQR)	40	124.0 (111.0,	123.0 (110.0,	123.0 (110.0,	123.0 (110.0,	123.0 (110.0,	123.0 (110.0,

	(2.4)	138.0)	137.0)	137.0)	137.0)	137.0)	137.0)

BMI, body mass index; e*GFR,* estimated glomerular filtration rate; *IQR,* interquartile range.

Table S3

Baseline characteristics of study participants by follow-up status (N = 2,160).

Characteristics	Successfully followed up (n=1,673)	Lost to follow-up (n=487)	P value [a]
Age (years), median (IQR)	87.00 (76.00, 99.00)	84.00 (73.00, 94.00)	<0.001
Female, no. (%)	930 (55. 6)	258 (53.0)	0.33
Married, no. (%)	631 (37.7)	220 (45.2)	0.004
Rural area, no. (%)	1,404 (83.9)	392 (80.5)	0.09
Living with a family member(s), no. (%)	1,293 (77.3)	366 (75.2)	0.36
Smoking status, no. (%)			<0.001
Current	271 (16.2)	92 (18.9)	
Former	138 (8.2)	103 (21.1)	
Never	1,264 (75.6)	292 (60.0)	
Drinking status, no. (%)			<0.001
Current	256 (15.3)	72 (14.8)	
Former	104 (6.2)	78 (16.0)	
Never	1,313 (78.5)	337 (69.2)	
Regular exercise, no. (%)			<0.001
Current	232 (13.9)	100 (20.5)	
Former	43 (2.6)	86 (17.7)	
Never	1,398 (83.6)	301 (61.8)	
BMI (kg/m^2), no. (%)			0.69
<18.5	448 (26.8)	118 (24.2)	
18.5-24	895 (53.5)	273 (56.1)	
24-28	253 (15.1)	75 (15.4)	
≥28	77 (4.6)	21 (4.3)	
Total sleep time (h), no. (%)			0.41
<6	293 (17.5)	98 (20.1)	
6-9	1,015 (60.7)	288 (59.1)	
>9	365 (21.8)	101 (20.7)	
Chronic disease, no. (%)			0.42
None	1,025 (61.3)	278 (57.1)	
One chronic disease	470 (28.1)	150 (30.8)	
Two chronic diseases	143 (8.5)	47 (9.7)	
Three or more chronic diseases	35 (2.1)	12 (2.5)	
Annual household income (yuan), no. (%)			<0.001
<10,000	709 (42.4)	167 (34.3)	
10,000-30,000	566 (33.8)	152 (31.2)	
>30,000	398 (23.8)	168 (34.5)	
Season of blood draw, no. (%)			0.003
Spring (March-May)	451 (27.0)	114 (23.4)	
Summer (June-August)	1142 (68.3)	331 (68.0)	
Autumn (September-November)	80 (4.8)	42 (8.6)	
25(OH) D (nmol/L), median (IQR)	36.90 (26.83, 51.46)	44.43 (33.80, 57.39)	<0.001
Albumin (g/L), median (IQR)	40.10 (36.90, 43.30)	40.20 (36.55, 43.55)	0.95
eGFR (ml/min per 1.73 m^2), no. (%)			0.34
≥90	4 (0.2)	1 (0.2)	
60-90	31 (1.9)	11 (2.3)	

30-60	359 (21.5)	125 (25.7)	
15-30	897 (53.6)	242 (49.7)	
<15	382 (22.8)	108 (22.2)	
Hemoglobin (g/L), median (IQR)	123.00 (110.00, 137.00)	121.00 (108.00, 133.50)	0.02

Notes: [a] P-values for continuous and categorical variables were respectively calculated using Kruskal-Wallis and chi-square tests. *BMI,* body mass index; *25(OH)D,* 25-Hydroxyvitamin D; e*GFR,* estimated glomerular filtration rate; *IQR*, interquartile range.

Table S4

Sensitive analysis of the associations between vitamin D concentrations and cognitive impairment after excluding participants with missing covariate data (N = 1,468)

25(OH)D (nmol/L)	Unadjusted Model	Model 1	Model 2
	OR (95% CI)	OR (95% CI)	OR (95% CI)
Cut-off at 50 nmol/L			
Low vitamin D (<50)	5.12 (3.50, 7.74) ***	3.48 (2.28, 5.47) ***	3.00 (1.89, 4.92) ***
Normal vitamin D (≥50)	Reference	Reference	Reference
Cut-offs by quartiles			
Quartile 1 (<27.1)	11.15 (7.18, 17.98) ***	5.96 (3.64, 10.05) ***	4.91 (2.86, 8.70) ***
Quartile 2 (27.1-37.9)	4.34 (2.75, 7.07) ***	2.87 (1.73, 4.88) ***	2.65 (1.50, 4.82) ***
Quartile 3 (37.9-52.4)	2.76 (1.71, 4.58) ***	2.68 (1.58, 4.66) ***	2.54 (1.47, 4.53) ***
Quartile 4 (≥52.4)	Reference	Reference	Reference

Notes: Model 1 adjusted for age and sex; model 2 further adjusted for the season of blood draw, living arrangement, marital status, drinking status, smoking status, regularity of exercise, body mass index, estimated glomerular filtration rate, hemoglobin, and albumin concentration. $^{*}P < 0.05$, $^{**}P < 0.01$, $^{***}P < 0.001$. *25(OH)D*, 25-Hydroxyvitamin D; *OR,* odd ratio; *CI,* confidence interval.

Table S5

Sensitive analysis of the individual associations of vitamin D concentrations and cognitive status with all-cause mortality after excluding participants with missing covariate data (N = 1,468)

Characteristics	Mortality, no. (per 100 person-year, %)	Unadjusted Model	Model 1	Model 2
		HR (95% CI)	HR (95% CI)	HR (95% CI)
25(OH)D (nmol/L)				
Cut-off at 50 nmol/L				
Low vitamin D (<50)	624 (16.4)	2.16 (1.80, 2.60) ***	1.71 (1.42, 2.06) ***	1.69 (1.37, 2.08) ***
Normal vitamin D (≥50)	140 (7.6s)	Reference	Reference	Reference
Cut-offs by quartiles				
Quartile 1 (<27.1)	257 (24.0)	3.20 (2.59, 3.96) ***	2.05 (1.64, 2.56) ***	2.20 (1.71, 2.82) ***
Quartile 2 (27.1-37.9)	211 (15.1)	1.99 (1.60, 2.48) ***	1.56 (1.25, 1.78) ***	1.58 (1.24, 2.01) ***
Quartile 3 (37.9-52.4)	170 (11.3)	1.49 (1.18, 1.87) ***	1.42 (1.12, 1.78) **	1.41 (1.10, 1.81) **
Quartile 4 (≥52.4)	126 (7.6)	Reference	Reference	Reference
P for trend		<0.001	<0.001	<0.001
Cognitive function				
Cognitive impairment	298 (36.9)	4.04 (3.48, 4.68) ***	1.77 (1.50, 2.09) ***	1.68 (1.42, 2.00) ***
Normal cognition	466 (9.7)	Reference	Reference	Reference

Notes: Model 1 adjusted for age and sex; model 2 further adjusted for the season of blood draw, living arrangement, marital status, drinking status, smoking status, regularity of exercise, body mass index, estimated glomerular filtration rate, hemoglobin, and albumin concentration, and further adjusted for cognitive status in vitamin D model, and 25(OH)D concentration in cognitive function model. $^{*}P < 0.05$, $^{**}P < 0.01$, $^{***}P < 0.001$. *25(OH)D*, 25-Hydroxyvitamin D; *HR,* hazard ratio; *CI,* confidence interval.

Table S6

Sensitive analysis of the combined associations of vitamin D concentrations and cognitive impairment with all-cause mortality after excluding participants with missing covariate data (N = 1,468)

Groups	Mortality, no. (per 100 person-year, %)	Unadjusted Model	Model 1	Model 2
		HR (95% CI)	HR (95% CI)	HR (95% CI)
Low vitamin D and cognitive impairment	274 (37.9)	11.81(7.89, 18.24) ***	6.83 (4.36, 11.04) ***	2.97 (2.29, 3.85) ***
Normal vitamin D and cognitive impairment	24 (28.0)	4.56 (3.00, 7.12) ***	3.29 (2.06, 5.37) ***	1.95 (1.23, 3.10) **
Low vitamin D and normal cognition	350 (11.4)	3.11(2.02, 4.91) ***	3.17 (1.96, 5.26) ***	1.74 (1.38, 2.18) ***
Normal vitamin D and normal cognition	116 (6.6)	Reference	Reference	Reference

Model 1 adjusted for age and sex; model 2 further adjusted for the season of blood draw, living arrangement, marital status, drinking status, smoking status, regularity of exercise, body mass index, estimated glomerular filtration rate, hemoglobin, and albumin concentration. $^{*}P < 0.05$, $^{**}P < 0.01$, $^{***}P < 0.001$. *HR,* hazard ratio; *CI,* confidence interval.
